# Eurasian-Origin Gene Segments Contribute to the Transmissibility, Aerosol Release, and Morphology of the 2009 Pandemic H1N1 Influenza Virus

**DOI:** 10.1371/journal.ppat.1002443

**Published:** 2011-12-29

**Authors:** Seema S. Lakdawala, Elaine W. Lamirande, Amorsolo L. Suguitan, Weijia Wang, Celia P. Santos, Leatrice Vogel, Yumiko Matsuoka, William G. Lindsley, Hong Jin, Kanta Subbarao

**Affiliations:** 1 Laboratory of Infectious Diseases, National Institute of Allergy and Infectious Diseases, National Institutes of Health, Bethesda, Maryland, United States of America; 2 MedImmune, Mountain View, California, United States of America; 3 National Institute for Occupational Safety and Health, Morgantown, West Virginia, United States of America; Erasmus Medical Center, Netherlands

## Abstract

The epidemiological success of pandemic and epidemic influenza A viruses relies on the ability to transmit efficiently from person-to-person via respiratory droplets. Respiratory droplet (RD) transmission of influenza viruses requires efficient replication and release of infectious influenza particles into the air. The 2009 pandemic H1N1 (pH1N1) virus originated by reassortment of a North American triple reassortant swine (TRS) virus with a Eurasian swine virus that contributed the neuraminidase (NA) and M gene segments. Both the TRS and Eurasian swine viruses caused sporadic infections in humans, but failed to spread from person-to-person, unlike the pH1N1 virus. We evaluated the pH1N1 and its precursor viruses in a ferret model to determine the contribution of different viral gene segments on the release of influenza virus particles into the air and on the transmissibility of the pH1N1 virus. We found that the Eurasian-origin gene segments contributed to efficient RD transmission of the pH1N1 virus likely by modulating the release of influenza viral RNA-containing particles into the air. All viruses replicated well in the upper respiratory tract of infected ferrets, suggesting that factors other than viral replication are important for the release of influenza virus particles and transmission. Our studies demonstrate that the release of influenza viral RNA-containing particles into the air correlates with increased NA activity. Additionally, the pleomorphic phenotype of the pH1N1 virus is dependent upon the Eurasian-origin gene segments, suggesting a link between transmission and virus morphology. We have demonstrated that the viruses are released into exhaled air to varying degrees and a constellation of genes influences the transmissibility of the pH1N1 virus.

## Author Summary

Influenza A viruses spread rapidly from person-to-person via respiratory droplets (RDs). In this study we used a ferret model to explore viral functions involved in RD transmission of influenza viruses. The 2009 pandemic H1N1 (pH1N1) virus originated by reassortment of a North American triple reassortant swine (TRS) virus with a Eurasian swine virus. Both TRS and Eurasian swine viruses had previously caused sporadic infections in humans, but failed to spread from person-to-person, unlike the pH1N1 virus. We evaluated the release of influenza virus-containing aerosols and the transmissibility of the pH1N1, TRS, and Eurasian viruses in ferrets and found that the Eurasian-origin gene segments contributed to efficient RD transmission of the pH1N1 virus by modulating the release of influenza viral RNA-containing particles into the air. The increased release of viral RNA-containing particles correlated with increased viral neuraminidase activity and production of filamentous viral particles. These observations enhance what we currently know about the viral requirements for influenza virus RD transmission and have implications for assessing the potential of novel influenza viruses to spread.

## Introduction

Influenza A viruses pose a global threat to human health. They circulate in animal hosts and can reassort to generate a virus to which the human population is naïve, creating a potential pandemic threat. Efficient person-to-person transmission of influenza A viruses via RDs is a feature of seasonal epidemics and of pandemics. Influenza viruses have caused several pandemics in the past, including one in 1918 caused by an avian-origin virus that killed 50 million people, and the most recent pandemic occurred in the spring of 2009 [1], [2]. The 2009 pandemic of swine-origin H1N1 influenza virus spread to over 215 countries from April 2009 to August 2010 and was responsible for at least 18,000 laboratory-confirmed deaths [3]. Determination of the molecular requirements for influenza viruses to transmit efficiently from person-to-person is an essential contribution to our understanding of potential pandemic threats. For example, the animal influenza viruses, avian H5N1, swine H1N1, and swine H1N2 viruses, have sporadically infected humans [4]–[8] but have not caused an influenza pandemic, presumably because they were unable to transmit efficiently throughout the human population.

The influenza A virus genome consists of 8 negative strand RNA gene segments that encode at least 11 proteins. The viral envelope is predominantly composed of the hemagglutinin (HA), neuraminidase (NA), and matrix (M1 and M2) proteins. HA is responsible for receptor binding and viral entry into a cell, while NA aids in release from the infected cell by cleaving sialic acids on the cell surface. The M1 protein lines the inside of the plasma membrane enveloping the viral RNA and gives structure to the virion, while M2 is an ion channel important for uncoating of the virus in the endosome and for virus release [9], [10]. The segmented genome allows reassortment to occur in nature, enhancing the genetic diversity of the virus. The 2009 pandemic H1N1 (pH1N1) virus arose from a reassortment event between a North American triple reassortant swine virus (TRS) and a Eurasian swine virus. The Eurasian swine viruses contributed the NA and M gene segments to the pH1N1 strain, while the remaining 6 gene segments came from the TRS virus [7], [11]. The pH1N1 precursor viruses, TRS and Eurasian swine, have transmitted from pigs to humans sporadically but secondary human cases did not occur [5], [8]. Recent studies have attempted to identify the genetic requirements for transmission of the pH1N1 virus [12], [13]. However, they did not identify the biological mechanisms by which these gene segments confer efficient transmission. Therefore, the biological determinants responsible for transmission of the pH1N1 virus that are lacking in the TRS and Eurasian swine viruses are still unknown.

Transmission of influenza virus has been studied extensively in animal models such as guinea pigs and ferrets [14], yet the precise mechanism or requirements for transmission are still unclear. Previous studies have suggested that host-range determinants such as receptor binding specificity and human-specific PB2 amino acid residues are important for transmission [15]–[19]. However, recent studies have demonstrated that these host-range determinants are not sufficient for transmission [20], [21]. Additionally, the HA protein from both the pH1N1 and TRS viruses is from the classical swine lineage that binds α2,6-linked sialic acids and both of these viruses contain avian-specific amino acids 627 and 701 in the PB2 gene, suggesting that those characteristics alone do not determine the transmissibility of these viruses. These observations suggest a role for other gene products in the transmissibility of the pH1N1 virus.

Three modes of influenza virus transmission have been defined: contact transmission, droplet spray transmission, and aerosol transmission. Contact transmission includes direct or indirect contact with a contaminated surface. Droplet spray transmission refers to person-to-person transmission via larger droplets that are deposited onto mucous membranes of the upper respiratory tract. Aerosol transmission is person-to-person transmission via aerosols composed of small, respirable particles that can be inhaled into the lower respiratory tract. The relative contribution of these different modes of transmission to person-to-person spread of influenza viruses is not known. In our study, the term respiratory droplet (RD) transmission includes both droplet spray and aerosol transmission. Studies attempting to distinguish between large and small aerosols have used aerosol samplers to measure the size of influenza virus-containing particles released by humans. Bio-aerosol sampling has been performed in various environmental settings such as hospitals, airplanes, and daycare centers [22]–[25]. These studies suggest that humans predominantly release small respirable particles that contain influenza virus, although larger particles containing influenza virus were also detected.

There are three components to consider when studying RD transmission of influenza virus: the donor, the environment, and the recipient. The donor must be infected with a virus that replicates efficiently in the upper respiratory tract and infectious virus must be released into the surrounding air. Environmental factors can alter the size, morphology, and amount of influenza virus-containing particles present in the air that is shared by the donor and recipient [26]. Recipients must be susceptible to viral infection and exposed to enough infectious virus to establish a productive infection. Modulation of any of these parameters, including viral host-range determinants, severity of disease symptoms, environmental temperature, humidity, and susceptibility of the recipient can alter the transmissibility of a virus [27], [28].

In this study, we used viruses generated by reverse genetics and biological isolates from human infections to explore the impact of the Eurasian-origin NA and M gene segments on transmissibility of the 2009 pH1N1 virus in a ferret model. We included the pH1N1 virus, representative Eurasian and TRS viruses that are putative precursor viruses, and a reassortant pH1N1 virus in which the NA and M gene segments were replaced with corresponding gene segments from a TRS virus. We found that the Eurasian NA and M gene segments contribute to efficient transmission of the pH1N1 virus. We used cyclone-based aerosol samplers to assess the amount and size distribution of influenza viral RNA-containing particles released by infected ferrets and determined the susceptibility of ferrets to the pH1N1 and its precursor viruses. Ferrets infected with viruses containing the Eurasian-origin NA and M gene segments efficiently released influenza viral RNA-containing particles into the air; this release correlated with higher NA activity of the pH1N1 and Eurasian viruses. Eurasian gene segments also contribute to the pleomorphic phenotype of the pH1N1 virus and this correlated with efficient RD transmission, suggesting a constellation of genes was responsible for the release of influenza virus-containing aerosols and transmissibility of the pH1N1 virus.

## Results

### Eurasian-Origin Gene Segments Confer Increased Transmission of pH1N1 Virus

RD transmission of pH1N1 virus has been shown to be highly efficient in the ferret model, with transmission efficiency ranging from 66% to 100% [29]–[31]. To assess whether the Eurasian-origin gene segments contribute to this phenotype, we used reverse genetics to create a recombinant pH1N1 virus and a 6∶2 reassortant pH1N1 virus in which the Eurasian-origin NA and M gene segments were replaced with the North American TRS NA and M gene segments (Table 1). We confirmed that the recombinant wild-type (wt) 2009 pH1N1 virus rescued by reverse genetics behaved similarly to the biological wt virus *in vitro* and *in vivo* (Figure S1). The titer of biological pH1N1 and recombinant pH1N1 viruses differed in the lungs of ferrets on day 1 (Figure S1B); however, by day 5 post-infection, viral replication in the lungs was equivalent. Therefore, we used the recombinant 2009 pandemic virus (rec A/California/07/2009), hereafter referred to as Rec pH1N1, as a surrogate for the biological virus in further studies.

**Table 1:** Genotype of the viruses.

			Origin								
Virus	Derived	Abbr Name	PB2	PB1	PA	HA	NP	NA	M	NS	Reference
rec A/CA/07/2009 V4 (H1N1)	Reverse genetics	Rec pH1N1	N. Am avian	Human H3N2	N. Am avian	CS	CS	ERAS	ERAS	CS	Chen Z et al 2010 [52]
rec A/CA/07/2009+A/OH/02/07 NA and M (H1N1)	Reverse genetics	6∶2 Reassort	N. Am avian	Human H3N2	N. Am avian	CS	CS	CS	CS	CS	This Study
A/OH/02/2007 (H1N1)	Biological isolate	TRS	N. Am avian	Human H3N2	N. Am avian	CS	CS	CS	CS	CS	Shinde et al 2009 [8]
A/Thailand/271/2005 (H1N1)	Biological isolate	Eurasian	ERAS	ERAS	ERAS	CS	ERAS	ERAS	ERAS	ERAS	Komadina et al 2007 [5]

Adapted from Garten et al 2009 Science [11].

Key: CS (Classical Swine); ERAS (Eurasian avian-like swine); N. Am (North American).

**Figure 1:**
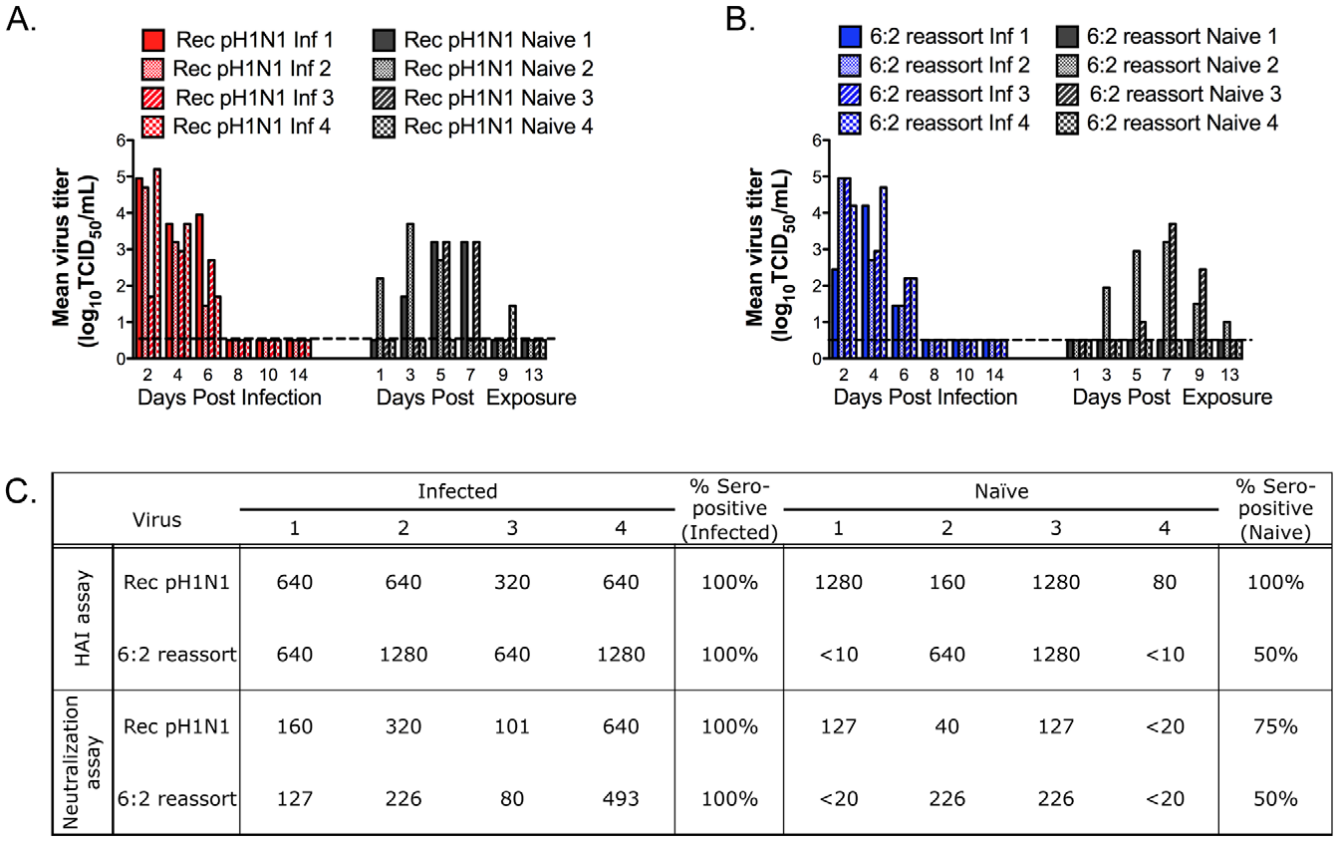
Eurasian-origin NA and M gene segments contribute to RD transmission of the pH1N1 virus. Four ferrets were inoculated IN to test the RD transmission of Rec pH1N1 (A) or the 6∶2 reassortant (B) viruses. Nasal washes were collected on the indicated days. Each bar represents the titer of virus from an individual ferret. Inf stands for infected ferret. The limit of detection is represented as the dashed line and is 10^0.5^ TCID_50_ per mL. Serum was collected on day 0 and day 14. Anti-influenza antibodies were measured by HAI and neutralization assay (C). The limit of detection is 1∶10 for HAI and 1∶20 for the neutralization assay. Antibody titers in the day 0 sera were below the limit of detection.

RD transmission studies were carried out in four independent transmission cages with the Rec pH1N1 virus and the recombinant A/California/07/2009+A/Ohio/02/2007 NA and M (6∶2 reassortant) virus. We measured viral titers in the nasal secretions of ferrets on alternate days for 14 days and determined levels of influenza-specific serum antibodies on day 14. A ferret was considered infected if it shed virus in the nasal secretions or seroconverted. We found that both the Rec pH1N1 and 6∶2 reassortant virus replicated to high titers in the nasal secretions of the infected ferrets (Figure 1A and B, left panels). Infected ferrets shed virus for 6 days, with a mean peak titer between 10^4.2^–10^5.2^ TCID_50_/mL on days 2 or 4 post-infection. All four of the naïve ferrets exposed to Rec pH1N1 virus shed infectious virus in the nasal secretions. Three of the naïve exposed ferrets shed virus from days 3 to 7 post-exposure, with a peak titer of 10^3.2^–10^3.7^ TCID_50_/mL of virus on day 5 for two of the ferrets (Figure 1A); this pattern of viral shedding is similar to data observed by us and others on the transmission of the biological pH1N1 virus (Figure S1D and [32]). The fourth ferret (naïve 4) shed virus much later than the other three (day 9 post-exposure). Presence of influenza-specific antibodies was found in all ferrets that shed virus in the nasal secretions (Figure 1C). Antibody titers for naïve ferret 4 were lower compared to the other ferrets, most likely because of the late onset of viral shedding. Since virus was detected in all 4 naïve ferrets and they all seroconverted by HAI, we concluded that RD transmission efficiency for the Rec pH1N1 virus was 100% in our system.

RD transmission of the 6∶2 reassortant virus was less efficient; virus was detected in the nasal secretions of two out of four naïve ferrets (Figure 1B), with peak shedding of 10^3.2^–10^3.7^ TCID_50_/mL on day 7 post-exposure. Influenza-specific antibodies were detected in the ferrets that shed virus in their nasal secretions (Figure 1C). These data demonstrate that replacement of the Eurasian-origin gene segments in the 6∶2 reassortant virus resulted in reduced transmission efficiency.

Additionally, we observed severe weight loss in two out of four ferrets infected with Rec pH1N1 virus and three out of four ferrets infected with the 6∶2 reassortant virus (Table S1), indicating that disease severity, as measured by weight loss, does not correlate with efficiency of RD transmission. Sneezing was observed in one of four ferrets for both viruses. Interestingly, in each case the naïve partner became infected, suggesting that generation of aerosols by sneezing may enhance transmission.

### Transmission Efficiency of the Pandemic Precursor Viruses

To determine whether the reduced transmission efficiency between the Rec pH1N1 and the 6∶2 reassortant virus was due to the Eurasian-origin gene segments, we evaluated the transmission efficiency of the pandemic precursor viruses. These experiments were conducted with swine-origin viruses that were isolated from humans (Table 1): for the North American TRS, an isolate obtained from an adult male in Ohio in 2007 (A/Ohio/02/2007) [8] and for the Eurasian swine virus, a virus isolated from a child in Thailand in 2005 (A/Thailand/271/2005) [5]. Both of these viruses had transmitted from pigs to humans, but did not spread from person-to-person [5], [6], [8].

**Figure 2:**
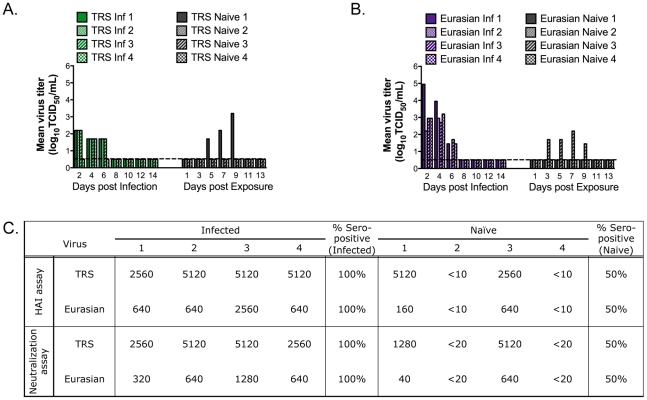
Pandemic precursor viruses transmit to 50% of exposed ferrets by RD. Four ferrets were inoculated IN to test the RD transmission of TRS (A) or the Eurasian (B) viruses. Nasal washes were collected on the indicated days. Each bar represents the titer of virus from an individual ferret. Inf stands for infected ferret. The limit of detection is represented as the dashed line and is 10^0.5^ TCID_50_ per mL. Serum was collected on day 0 and day 14. Anti-influenza antibodies were measured by HAI and neutralization assay (C). The limit of detection is 1∶10 for HAI and 1∶20 for the neutralization assay. Antibody titers in the day 0 sera were below the limit of detection.

Ferrets infected with the A/Ohio/02/2007 (TRS) virus had much lower titers of virus in their nasal secretions (Figure 2A) than those infected with the Rec pH1N1 or 6∶2 reassortant viruses. The peak titer was 10^2.2^ TCID_50_/mL for most ferrets on day 2-post infection. However, in other experiments performed in our lab and by others, this virus replicated to higher levels in the upper respiratory tract of ferrets (Table 2, Figure S1A and [32]). Ferrets infected with the A/Thailand/271/2005 (Eurasian) virus shed high titers of the virus (Figure 2B). Peak viral shedding was observed on days 2 or 4 post- infection, with peak titers of 10^2.95^–10^4.95^ TCID_50_/mL. A matched non-parametric two-way ANOVA of the nasal wash titers in animals infected with Eurasian, pH1N1, or 6∶2 reassortant virus showed no statistical difference among these groups of viruses.

**Table 2:** Infectivity of pH1N1 influenza and precursor viruses for ferrets.

Virus	Dose (TCID_50_) of virus administered^a^	No. seroconverted/total^b^	No. shedding virus (Culture pos/total)	50% ferret infectious dose (FID_50_)^c^	Mean peak titer in nasal wash (log_10_ TCID_50_/mL)
	10	1/3	1/3		3.2
Rec pH1N1	100	3/3	3/3	18	2.4
	10,000	3/3	3/3		4.2
	10	1/3	2/3		3.45
6∶2 Reassort	100	3/3	3/3	18	4.95
	10,000	3/3	3/3		3.7
	10	1/3	1/3		4.45
TRS	100	3/3	3/3	18	3.7
	10,000	3/3	3/3		4.45
	10	3/3	3/3		4.3
Eurasian	100	3/3	3/3	<10	3.7
	10,000	3/3	3/3		3.6

a Virus dose delivered in 500 µL volume.

b Seroconversion was determined by HAI assay.

c If the endpoint was not reached at a dose of 10 TCID_50_, we assumed that at a dose of 1 TCID_50_ no ferrets would be infected; therefore, the FID_50_ value is shown as <10.

One of four ferrets each infected with either the TRS or Eurasian viruses developed a distinctive cough, similar to croup (Table S1). The naïve ferrets paired with the croupy ferret became infected with influenza and shed virus in their nasal secretions, suggesting that the aerosols released by coughing enhanced RD transmission. These were the only ferrets that shed virus in the nasal secretions after exposure to ferrets infected with the TRS or Eurasian viruses. As seen with the Rec pH1N1 and 6∶2 reassortant viruses, all of the ferrets with detectable virus in the nasal secretions also produced anti-influenza antibodies (Figure 2C). However, with both the TRS and Eurasian viruses, one naive ferret (TRS naïve 3 and Eurasian naïve 1) that did not shed virus in the nasal secretions seroconverted. Others have also found serologic evidence of infection in the absence of virologic evidence in a ferret transmission model [33]. Thus, we conclude that the two pandemic precursor viruses transmitted with 50% efficiency in ferrets. Our data demonstrate that the Eurasian-origin NA and M gene segments are necessary, but not sufficient, for RD transmission in our ferret model. To confirm that the reduced RD transmission of the TRS (A/Ohio/02/2007) was not due to the lower viral replication in the experimentally infected ferrets, we re-evaluated the replication and transmissibility of this virus with a larger number of animals. We confirmed the earlier finding of reduced transmissibility, even in the face of higher titers of virus in the experimentally infected ferrets. The TRS virus replicated to variable titers in the nasal secretions of experimentally infected ferrets; some infected ferrets had low titers (10^1.7^–10^2.95^ TCID_50_/mL), consistent with the titers we had observed in the first study (Figure 2A) and others had higher titers (10^3.7^–10^3.95^ TCID_50_/mL) of virus (Figure S2A). The peak of viral shedding was on day 2, as previously observed. In the new transmission study, only 2 out of 6 naïve animals became infected, as defined by isolation of virus in their nasal secretions and/or seroconversion (Figure S2B). The reduced transmission efficiency of the TRS virus has also been reported by others [13], [32]. Additionally, since ferrets infected with the pH1N1, 6∶2 reassortant, or Eurasian virus all shed virus to similar levels, we believe that RD transmission is not dependent upon efficient virus replication in the nasal secretions of animals. Therefore, efficient RD transmission is likely due to other factors such as infectivity of the virus for the naïve host or release of viral particles into the air.

### Infectivity of the Pandemic and Precursor Viruses for Ferrets

To determine whether the infectivity of the viruses for ferrets varied, we determined the dose of virus at which 50% of ferrets were infected (FID_50_). Ferrets were inoculated with 10,000, 100, or 10 TCID_50_ of virus, and infectivity was measured by the presence of infectious virus in nasal secretions or by seroconversion. Table 2 lists the number of ferrets at each dose that were infected among the ferrets that were inoculated with each dose. Peak virus titers obtained from the nasal secretions are also presented in Table 2. In this experiment, ferrets infected with the TRS virus shed virus in the nasal wash at titers equivalent to the other viruses, confirming that this virus has variable replication in the upper respiratory tract of ferrets. Interestingly, administration of doses of virus as low as 10 TCID_50_ resulted in peak viral titers similar to that of 1000-fold higher doses. Based on the data presented in Table 2, Rec pH1N1 and TRS viruses have a similar FID_50_, and the 6∶2 reassortant and Eurasian viruses are more infectious. Surprisingly, all 3 animals infected with 10 TCID_50_ of the Eurasian virus shed virus in nasal washes and seroconverted (Table 2). These data demonstrate that while the pandemic virus and its precursors may differ in their infectivity in ferrets, this does not correlate with transmissibility of these viruses via RD transmission.

### Release of Influenza Viral RNA-containing Particles into the Air Depends on the Presence of the Eurasian-Origin Gene Segments

**Figure 3:**
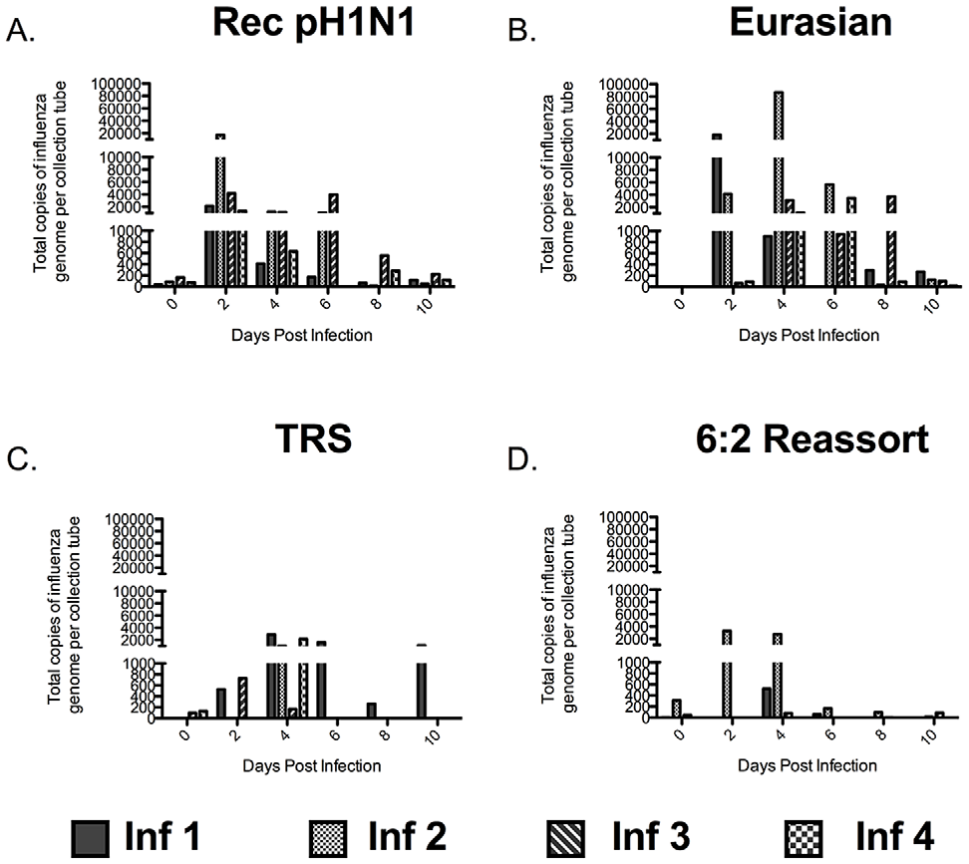
The Eurasian-origin NA and M gene segments contribute to abundant release of large (>4 µm) particles containing influenza virus. Quantitative (Q)-PCR for influenza A M gene in RNA extracted from the 15 mL collection tube of the cyclone-based air samplers. Air was collected for one hour on the outside of the infected ferret cage. Each bar represents the amount of genome copies of influenza in particles released by a single ferret infected with Rec pH1N1 (A), Eurasian (B), TRS (C), or 6∶2 reassortant (D). Absolute amount of RNA was quantitated using a standard curve of in vitro transcribed influenza M gene RNA. Inf stands for infected ferret.

**Figure 4:**
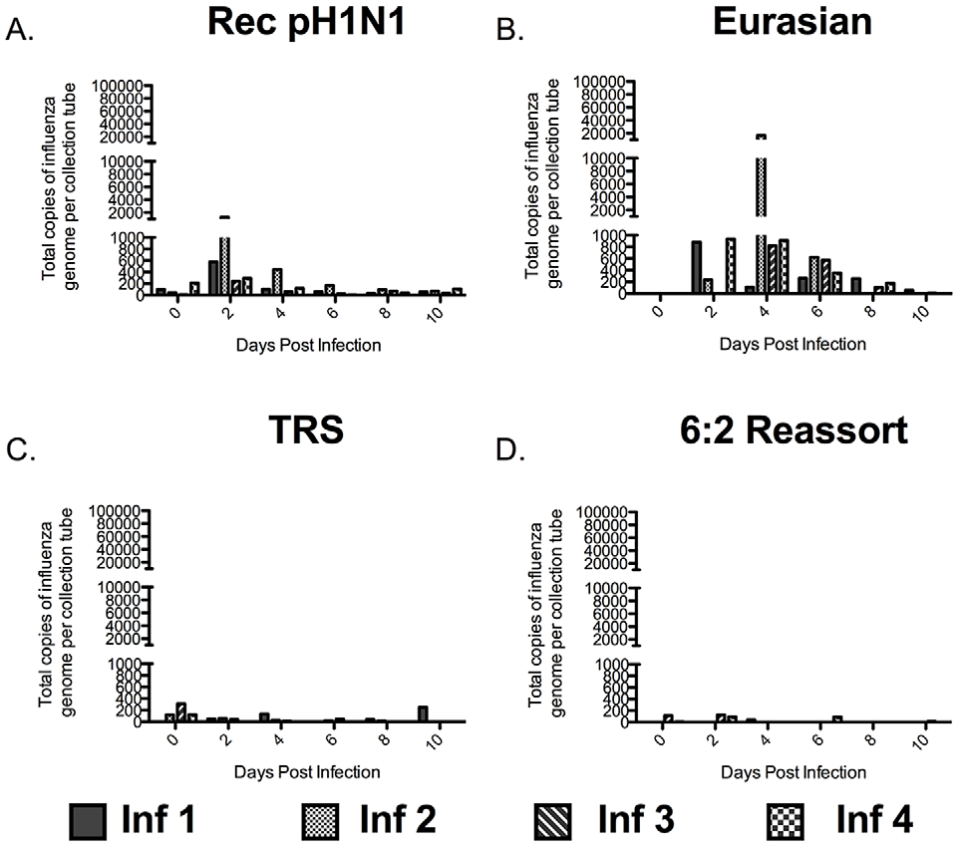
The Eurasian-origin NA and M gene segments contribute to the abundant release of 1 to 4 µm particles containing influenza virus. Q-PCR for influenza A M gene on RNA extracted from the 1.5 mL collection tube of the cyclone-based air samplers. Air was collected for one hour on the outside of the infected ferret cage, each bar represents the amount of particles released by a single ferret infected with Rec pH1N1 (A), Eurasian (B), TRS (C), or 6∶2 reassortant (D). Absolute RNA was quantitated using a standard curve of in vitro transcribed influenza M gene RNA. Inf stands for infected ferret.

**Figure 5:**
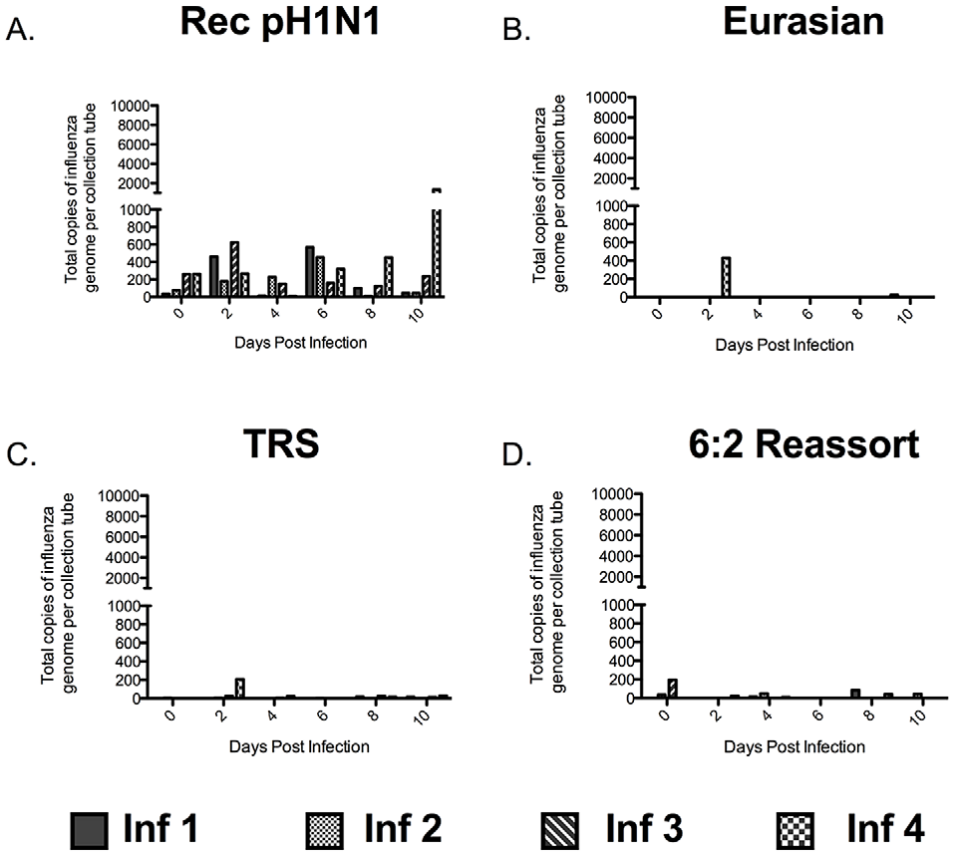
Ferrets infected with the recombinant pH1N1 virus release submicron particles containing influenza virus. Q-PCR for influenza A M gene on RNA extracted from the filter of the cyclone-based air samplers. Air was collected for one hour on the outside of the infected ferret cage, each bar represents the amount of particles released by a single ferret infected with Rec pH1N1 (A), Eurasian (B), TRS (C), or 6∶2 reassortant (D). Absolute RNA was quantitated using a standard curve of in vitro transcribed influenza M gene RNA. Inf stands for infected ferret.

Influenza virus particles must be released into the air for RD transmission to occur. Much work has been done recently exploring the size distribution of particles containing influenza virus that are released by humans [22]–[24], [34], [35]. However, few studies have been done in animal models to correlate the amount of particles released with influenza virus transmission [36], [37]. To determine the size of influenza virus particles in the air exhaled by infected ferrets, we used cyclone-based aerosol samplers that separate particles based on size; these samplers have previously been used in clinical settings to assess exposure of health care workers to influenza [22], [23]. The samplers have three collection surfaces: a 15 mL conical tube captures particles greater than 4 µm, a 1.5 mL tube captures particles between 1 and 4 µm, and a filter traps all submicron (<1 µm) particles. The samplers were secured to the outside of the cage, between the inner and outer doors (Figure S3A), and the air on the infected ferret's side of the cage was sampled for one hour on alternate days at a rate of 3.5 liters per minute. The ferrets were undisturbed during air sampling. The distribution of influenza viral RNA-containing particles released by infected ferrets was determined for each virus in the study at all 3 sizes: >4 µm (Figure 3), 1 to 4 µm (Figure 4), and <1 µm (Figure 5). This system does not allow for the measurement of the total count of particles released by each ferret nor the isolation of infectious virus because the collection tubes are dry. However, it does allow for the quantification of particles containing influenza viral RNA. We used this measurement as a surrogate for the amount of viral particles present in aerosols of various sizes. We found that ferrets predominantly released influenza virus into the air in large particles (>4 µm) (Compare Figures 3, 4, and 5). The duration for which the large particles containing influenza viral RNA was detected correlated with the length of time that virus was detected in the nasal washes of the ferrets. In the case of the Rec pH1N1 virus, all four infected ferrets consistently released large particles containing influenza viral RNA for 6 days post-infection, with a peak on day 2 (Figure 3A). Although virus was not detected in the nasal wash on day 8 post-infection, a low level of aerosol particles containing influenza viral RNA was observed. Ferrets infected with the Eurasian swine virus also consistently released influenza viral RNA-containing particles into the air, and in a larger quantity than the Rec pH1N1 infected ferrets (Figure 3B). Ferrets infected with the 6∶2 reassortant and TRS viruses sporadically released large (>4 µm size) influenza viral RNA-containing particles (Figure 3C and D). To compare the trend of influenza viral RNA-containing particles released by animals infected with these viruses, we calculated the average area under the curve (AUC) for each virus per collection tube. We found that AUC of the Rec pH1N1 and Eurasian viruses for the 15 mL collection tube are 5540 and 29,384 respectively. These values are higher than those for the 6∶2 reassortant and TRS viruses, which are 1338 and 2333, respectively. These data demonstrate that while ferrets predominantly released large influenza viral RNA-containing particles, the ferrets infected with Rec pH1N1 and Eurasian viruses released more than those infected with either the TRS or 6∶2 reassortant virus. A similar phenomenon was found with the release of 1 to 4 µm-sized particles (Figure 4). Viruses containing the Eurasian-origin gene segments (Rec pH1N1 and Eurasian) also had a more consistent release of influenza viral RNA-containing particles at the 1–4 µm size (Figure 4A and B), while the TRS and 6∶2 reassortant virus had a more sporadic release of influenza viral RNA-containing particles (Figure 4 C and D). This phenomenon was confirmed by analysis of the average AUCs for each respective graph; the Rec pH1N1 and Eurasian viruses had AUC values (636.5 and 5464, respectively) higher than the TRS and 6∶2 reassortant viruses (124.8 and 59.3, respectively). Ferrets infected with pH1N1 virus released 1–4 µm particles containing influenza viral RNA from day 2 to 4 with a peak at day 2, while some animals infected with the Eurasian virus released these particles consistently on days 2, 4, and 6. Very few influenza viral RNA-containing particles were detected 6 days post-infection (Figure 4). Although it is possible that the sporadic release of influenza viral RNA-containing particles from ferrets infected with the TRS virus may be linked to the low viral titers in the nasal secretions (Figure 2A), a similar pattern of sporadic release was also seen in the repeat experiment of ferrets infected with the TRS virus (Figure S2C). Additionally, ferrets infected with the 6∶2 reassortant virus shed virus to high titers in the nasal secretions and also displayed a sporadic release of particles containing influenza viral RNA. Therefore, we conclude that the Eurasian-origin gene segments contribute to the release of influenza viral RNA-containing particles greater than 1 µm.

Interestingly, the pattern of release of submicron particles containing influenza viral RNA by the Rec pH1N1 virus was different from the other viruses (Figure 5 A and B). Ferrets infected with the Rec pH1N1 virus consistently released submicron particles containing influenza viral RNA into the air, and this release was detected at every time point tested, with a similar amount on days 2 and 6 post-infection. Interestingly, infected ferret number 4 released a considerable amount of submicron particles containing influenza viral RNA on days 8 and 10 post-infection, which correlates with the late infection of its naïve pair (refer to Figure 1A). In contrast to the Rec pH1N1-infected ferrets, those infected with the Eurasian virus released submicron influenza viral RNA-containing particles only sporadically. Infection with the TRS and 6∶2 reassortant virus did not result in release of submicron influenza viral RNA-containing particles into the air that were detectable by our sampling system (Figure 5 C and D). There was a higher background observed in the Rec pH1N1 infected ferrets on day 0 compared to the other viruses that may be due to an environmental contaminant. Despite this, the release of influenza viral RNA-containing particles from ferrets infected with the Rec pH1N1 virus was found to be distinct from the other viruses. A comparison of the average AUC values from days 2 to 10 confirms this observation; the pH1N1 virus had an AUC value of 1043 and all of the other viruses had AUC values ranging from 56 to 59. Additionally, a two-way ANOVA found that the difference in the amount of submicron particles that contained influenza viral RNA released by ferrets infected with Rec pH1N1 virus compared with all other viruses was significant. The release of submicron influenza viral RNA-containing particles correlates with transmission efficiency and it is tempting to speculate that RD transmission is associated with these submicron particles.

Overall, our air sampling studies have found that ferrets infected with viruses that lacked the Eurasian-origin NA and M gene segments, the TRS and 6∶2 reassortant viruses, only sporadically released influenza viral RNA-containing particles of all sizes into the air (Figures 3, 4, and 5). This finding suggests that the Eurasian-origin gene segments contribute to the transmissibility of the pH1N1 virus by influencing the release of influenza viral RNA-containing particles into the air.

### Release of Influenza Viral RNA-containing Particles Correlates with NA Activity and Virus Morphology

**Figure 6:**
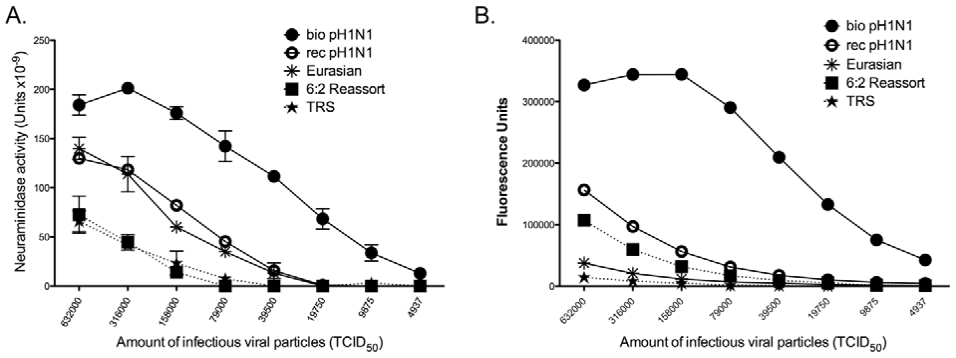
Viruses with Eurasian-origin NA have greater neuraminidase activity than viruses with a classical swine NA. An ELLA assay using fetuin as a substrate was used to determine the NA activity for the biological pH1N1 (•), rec pH1N1 (<$>\vskip -1\scale 70%\raster="rg1"<$>), 6∶2 reassortant (▪), TRS (★), and Eurasian (*) viruses (A). Neuraminidase activity of these viruses was also measured using MUNANA as a substrate (B). Viruses were normalized for equal infectivity in all assays. The data are displayed as an average of 2 independent assays performed in duplicate. Error bars represent the standard error.

The neuraminidase activity of the influenza NA protein cleaves sialic acids from the proteins on the cell surface and on the viral surface [9]. The cleavage of sialic acids by the viral neuraminidase aids in viral release and the prevention of viral agglutination after release. Therefore, it is plausible that infection with a virus with a more active NA could result in the release of more virus particles into the air. To determine whether the activity of the Eurasian-origin NA differs from that of the classical swine NA, we used an enzyme-linked lectin assay to determine the neuraminidase activity of viruses that had been normalized for infectivity using fetuin as a substrate (Figure 6A). Viruses that contain the Eurasian NA (the biological and recombinant pH1N1 and the Eurasian viruses) had higher NA activity than the TRS and 6∶2 reassortant viruses, which have a classical swine NA protein. A similar observation has been made previously using MUNANA as a substrate [13]. To confirm these results, we performed a neuraminidase assay using MUNANA as a substrate (Figure 6B). MUNANA and fetuin differ in size; MUNANA is a short α2,6-linked sialic acid substrate while fetuin is much larger and contains both α2,3- and α2,6-linked sialic acids [38], [39]. Since little is known about the biological substrates cleaved by NA in vivo, it is difficult to determine which substrates are biologically most relevant. We found that the Rec pH1N1 virus had a lower NA activity than the biological pH1N1 virus in both assays. The consensus sequence for the NA gene was identical for these viruses, suggesting that differences in the minor quasispecies composition of the respective virus populations may be the factor. Interestingly, with MUNANA, the Eurasian virus had lower NA activity than the pH1N1 virus, suggesting that NA proteins may have variable activity on different substrates. Our data indicate that the pH1N1 virus has a higher neuraminidase activity than the TRS and 6∶2 reassortant viruses with both long and short substrates, and higher neuraminidase activity than the Eurasian virus with short substrates. These observations suggest that NA activity correlates with the release of virus particles and increased viral release is important for efficient RD transmission of the pH1N1 virus.

**Figure 7:**
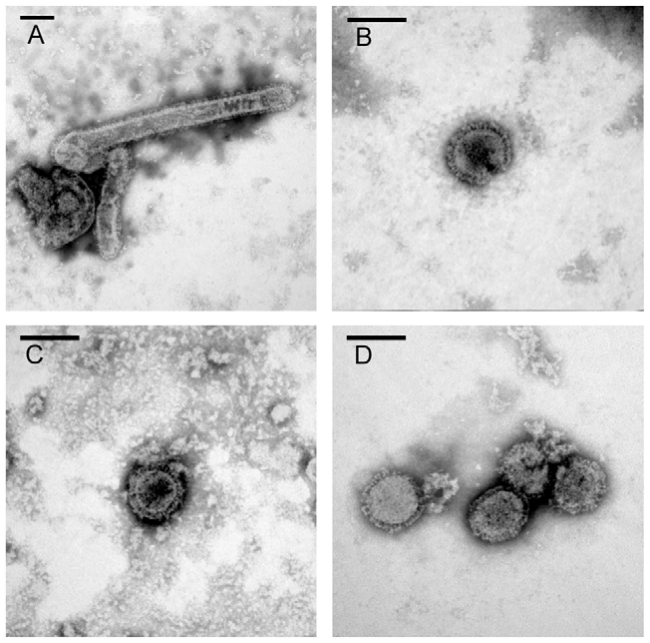
Eurasian-origin gene segments confer filamentous morphology of pH1N1 virus. Electron micrographs of negatively stained virus preparations are shown for Rec pH1N1 (A), 6∶2 reassortant (B), TRS (C), and Eurasian (D) viruses. Representative images are shown for each virus. Bar; 100 nm.

The Eurasian swine virus contributed both the NA and M gene segments to the pH1N1 virus and the M protein has been implicated in determining the filamentous or spherical morphology of influenza viruses [40]–[42]. Therefore, we compared the morphology of the Rec pH1N1, 6∶2 reassortant, Eurasian, and TRS viruses by electron microscopy (Figure 7). The pH1N1 virus has previously been reported to be pleomorphic [29] and similar morphology was observed for the Rec pH1N1 virus (Figure 7A). We counted 20 or more particles and found that 60% of the Rec pH1N1 virus particles were filamentous, while the 6∶2 reassortant virus was predominantly spherical with only 4% filamentous particles (Figure 7B). These data suggest that the Eurasian-origin gene segments specify the pleomorphic phenotype of the pH1N1 virus. The pH1N1 precursor viruses (Eurasian and TRS) were both predominantly spherical (Figure 7C and D), with only 9.5% or 0% filamentous particles, respectively. Taken together, these observations indicate that the Eurasian-origin gene segments alone are not sufficient to specify the pleomorphic morphology of the pH1N1 virus. The cytoplasmic tails of both HA and NA have previously been shown to contribute to influenza viral morphology [43]. However, the viruses used in this manuscript all contain the classical swine HA. Therefore, it is likely that specific adaptations in the pH1N1 viral gene segments that are distinct from the Eurasian swine gene segments have arisen and these changes may have contributed to the pleomorphic nature of the pH1N1 virus. Additionally, the complete passage history of the Eurasian virus is not known but may be relevant to its morphology.

Previous studies have suggested that receptor specificity correlates with RD transmission [17], [44]. However, all of the viruses tested in this study have HA proteins that are evolutionarily similar to the classical swine virus (Table 1) and are antigenically similar to each other (data not shown). We evaluated receptor binding specificity using an in vitro assay with chicken RBCs specifically sialylated with α2,3 or α2,6 sialyltransferases (Figure S4A) and demonstrated that all of the viruses predominantly associate with α2,6-linked sialic acids.

Since virus-receptor affinity may be altered during viral evolution [45], we tested whether the viruses used in this study differed in their affinity for the α2,6 receptor by measuring their ability to agglutinate chicken red blood cells (RBCs) that had been treated with varying amounts of neuraminidase (Figure S4B). We found that all of the viruses bound to RBCs that were desialylated with similar concentrations of bacterial neuraminidase; therefore, we conclude that neither receptor specificity nor receptor affinity are responsible for the particle release observed in this study.

Taken together, our data suggest a role for the Eurasian-origin segments in the morphology and NA activity of the pH1N1 virus, one or both of which contribute to its efficient transmission.

## Discussion

This study was designed to identify the molecular determinants that confer transmissibility of the pH1N1 virus and the mechanism by which they promote transmission. RD transmission can be modulated at the level of the infected donor, the environment, and the recipient. We established an RD transmission caging system that allowed for aerosol sampling of infected ferrets. In our system, the Rec pH1N1 virus transmitted to 100% of the naïve animals and replacement of the NA and M gene segments with the corresponding gene segments from TRS resulted in reduced transmission efficiency. These findings indicate that the Eurasian-origin NA and M gene segments contribute to the efficient transmission of the Rec pH1N1 virus. The fact that the Eurasian virus only transmitted to 50% of the naïve animals demonstrates that gene constellation may influence this phenotype as it does other properties such as virulence [46]. Yen et al. have recently suggested that a balance between HA and the Eurasian-origin NA contribute to the transmissibility of the pH1N1 virus [13]. Unlike our study, they used swine isolates that had not infected humans; therefore, any compensatory mutations that promote the initial transmission from an animal host to human were not taken into account. Based on our results, we believe that the biological properties of both Eurasian-origin gene segments influence particle release and thus efficient RD transmission. In our study, we found that susceptibility of the recipient ferrets to the specific virus, measured as the FID_50_ of the virus, did not correlate with transmission efficiency. Since environmental factors such as temperature and relative humidity were unaltered during the study, they did not contribute to the transmission phenotype. Therefore, we focused our attention on the release of influenza viruses by the infected donor ferrets. The viruses used in this study shared similar receptor specificity and replicated efficiently in the upper respiratory tract of ferrets. These two factors have been implicated in the transmissibility of other influenza viruses but they did not contribute to the enhanced transmission phenotype of the pH1N1 virus in our study. Using aerosol biosamplers to measure the release of virus into the air, we found that viruses containing the Eurasian-origin NA and M gene segments released influenza viral RNA-containing particles into the air consistently and this correlated with increased NA activity of these viruses. The Eurasian-origin gene segments also conferred the pleomorphic phenotype of the pH1N1 virus. Our observations extend our knowledge of the molecular determinants of RD transmission and provide an explanation for the epidemiological success of the pH1N1 virus.

An infected donor can generate aerosols during normal breathing or upon sneezing and coughing [47]. In our study, we used ferrets as donors because they are highly susceptible to influenza viruses and can both transmit the virus to humans and acquire infection from humans [48]. Ferrets infected with influenza viruses develop clinical symptoms such as weight loss, sneezing, and lethargy [49]. Disease severity in ferrets and humans varies by strain, with highly pathogenic strains such as H5N1 avian influenza viruses causing more severe disease than seasonal influenza strains [29]–[31]. We found that the 2009 pH1N1 virus and its precursor viruses caused similar disease severity in ferrets, defined by >10% weight loss and presence of clinical symptoms like sneezing and runny nose (Table S1). However, we also found that one out of four ferrets infected with TRS or Eurasian viruses developed croup and were able to efficiently transmit the virus to their naïve partners. Upon further analysis, we found a correlation between infected ferrets that were observed sneezing or coughing and infection of their naïve neighbors, indicating that generation of aerosols by sneezing and coughing enhances RD transmission.

In this study, we examined the size of influenza viral RNA-containing particles released from ferrets infected intranasally (IN) with influenza viruses and found that the ferrets primarily released influenza viral RNA-containing particles greater than 4 µm in size into the air (Figure 3). Consistent with our observations, Gustin et al. reported that anesthetized ferrets infected IN predominantly released large (>4.7 µm) infectious particles during normal breathing. However, they found that ferrets infected by aerosol released much smaller (0.65 to 4.7 µm) particles containing infectious virus into the air [37]. We found that ferrets inoculated IN with pH1N1 and Eurasian viruses released large (>4 µm) and small (1 to 4 µm) influenza viral RNA-containing particles more consistently than ferrets infected with the TRS and 6∶2 reassortant viruses (Figure 3 and 4). The viruses with more consistent release of virus had a higher NA activity than viruses that were associated with sporadic release of influenza viral RNA-containing particles (Figure 6). Thus, NA activity correlates with the release of both large and small influenza viral RNA-containing particles. However, these particles are not sufficient for efficient RD transmission since the Eurasian virus, which consistently released large and small influenza viral RNA-containing particles, transmitted to only 50% of the naïve animals (Figure 2B). Additionally, in animals infected with the TRS virus, we only detected the presence of large particles containing influenza viral RNA in the air, yet this virus transmitted to 50% of the naïve animals. These data suggest that the large particles (>4 µm) may contribute to RD transmission of viruses in the ferret model system. Release of large particles containing influenza has been observed in human clinical studies [23]. However, the relative importance of these particles in human transmission is unclear.

Interestingly, release of submicron influenza viral RNA-containing particles differed between pH1N1 and the Eurasian viruses (Figure 5). The Rec pH1N1 infected ferrets consistently released submicron influenza viral RNA-containing particles while ferrets infected with the Eurasian virus did not. Given that the animal cages have a continuous air flow rate of 40 cubic feet per minute, it is also possible that we were unable to thoroughly capture the submicron particles released by the ferrets by sampling on the outside of the cage. Aerosol sampling in different environments suggests that humans predominantly release small, respirable particles that likely result in the respiratory or aerosol transmission of influenza viruses [22], [23], [34]. Since the pH1N1 infected ferrets released more submicron particles than ferrets infected with any of the other viruses, it is possible that the submicron particles are responsible for the efficient aerosol transmission of the pH1N1 virus.

Previous studies have demonstrated a role for HA receptor binding specificity and specific amino acid residues in the PB2 protein on RD transmission of influenza A viruses [17]–[19], [50]. The emergence and transmissibility of the 2009 pH1N1 virus cannot be explained by these molecular determinants of transmissibility of the virus via RDs. Instead, our study illustrates the importance of the NA and M proteins in the transmissibility of the pH1N1 virus. We found that NA activity correlates with the release of particles greater than 1 µm in size and this may be necessary, but not sufficient, for RD transmission. Additionally, we found that viral morphology correlated with transmissibility of swine-origin viruses in the ferret model. The pleomorphic Rec pH1N1 virus was more efficiently transmitted than the spherical 6∶2 reassortant, TRS, and Eurasian viruses, suggesting that this phenotype may be important for RD transmission of swine-origin viruses. While there are many examples of α2,6-specific receptor binding influenza viruses that do not transmit in animal models or in the human population [14], [51], there are no reports of RD transmission of α2,3-specific receptor binding influenza viruses. Therefore, virus receptor binding specificity is also necessary, but not sufficient, for transmission.

Our data indicate that in order to more accurately assess pandemic threat potential, phenotypes that are important for transmission such as viral replication in the upper respiratory tract of ferrets, release of respirable influenza virus-containing particles, and receptor specificity of novel influenza viruses should be characterized.

## Materials and Methods

### Ethics Statement

This study was carried out in strict accordance with the recommendations in the Guide for the Care and Use of Laboratory Animals of the National Institutes of Health. The National Institutes of Health and MedImmune Animal Care and Use Committee (ACUC) approved the animal experiments that were conducted at the respective facilities. All efforts were made to minimize suffering.

### Cells and Viruses

Madin-Darby canine kidney (MDCK) cells, obtained from the ATCC, were maintained in minimum essential media (MEM) and 10% fetal bovine serum (FBS). 293T cells, obtained from ATCC, were maintained in Dulbecco's MEM with 10% FBS.

The reverse genetics system for generating the 2009 pandemic H1N1 virus (A/California/07/2009) were previously described [52]. The NA and M gene segments for the North American TRS virus (A/Ohio/02/2007) were constructed as previously described [52]. The recombinant viruses generated from the reverse genetics plasmids were rescued from MDCK/293T cell co-culture and propagated in specific pathogen free (SPF) embryonated eggs as described [53] for 2 passages. Viruses generated by reverse genetics were confirmed by genomic sequencing. The A/Ohio/02/2007 (H1N1) and A/Thailand/271/2005 (H1N1) viruses were obtained from the Centers for Disease Control and Prevention (CDC) and were subsequently propagated in MDCK cells. The passage histories for the biological isolates are C5 and CX,C3/C2/C2 for A/Ohio/02/2007 and A/Thailand/271/2005, respectively where X indicates an unknown number of passages.

### Ferret Infections and Nasal Wash Collection

All transmission studies consisted of four RD transmission cages, 3 male cages and 1 female cage. Each transmission cage contained two ferrets – 1 naïve and 1 infected, per cage (Figure S3). For each study 6 male and 2 female, 5–8 month old adult ferrets obtained from Triple F farms (Sayre, PA) that were seronegative for seasonal H3 and H1 viruses, and all of the viruses used in this study. As in other RD transmission studies [27], [33], [51] the sample size is small. Ferrets were inoculated intranasally (IN) with 10^6.5^ TCID_50_ of virus in 500 µL of Leibovitz-15 medium. All ferrets were monitored for clinical signs including sneezing, coughing, lethargy, weight loss, and body temperature changes. In accordance with NIAID Animal Care and Use Committee (ACUC) guidelines, ferrets were euthanized if they lost more than 20% of their initial body weight.

Ferret infectivity studies were performed at MedImmune (Mountain View, CA). Two male and one female adult ferrets (5–6 month old) were inoculated IN with each dose (10, 100, or 10,000 TCID_50_ per 500 µL) of virus. Ferrets were considered infected if one of the following criteria was met: detection of virus in nasal secretions or by the presence of >40 influenza-specific antibody titer in the sera. Ferret infectious dose 50 (FID_50_) values were calculated using the method described by Reed and Muench [54].

Nasal secretions were collected by washing the right nostril of an anesthetized ferret with sterile PBS and 500 µL of liquid that was expelled from the left nostril was collected. These nasal secretions were analyzed for the presence and titer of infectious viruses and expressed as 50% tissue culture infectious doses (TCID_50_) per mL.

### Transmission Studies

We designed the caging system for transmission studies based on earlier reports [33]. Briefly, large stainless steel ventilated ferret cages from Allentown (Allentown, New Jersey) were modified for the RD transmission studies (Figure S3). Two perforated stainless panels were welded together, 0.5 inches apart, and placed into the cage with a floor and ceiling guide to stabilize the panel. A door, with separate feeder and water bottles on each side of the dividing panel, was manufactured for each cage. Infected ferrets were placed into the section of the cage closest to the air inlet one day prior to infection. One day post-infection, a naïve ferret was placed into the cage on the other side of the divider. Environmental conditions inside the laboratory were monitored daily and were consistently 19±1°C and 56±2% relative humidity. The transmission experiments were conducted in the same room, to minimize any effects of caging and airflow differences on aerobiology. Nasal washes were collected and clinical signs were recorded on alternate days for 14 days. Air samples were collected between 9 am to 12 pm on alternate days for 10 days. On day 14 post-infection, blood was collected from each animal for serology. The naïve ferret was always handled before the infected ferret. Great care was taken during nasal wash collections and husbandry to ensure no direct contact occurred between the ferrets.

### Serology

Ferret sera were tested for the presence of anti-influenza antibodies by hemagglutination inhibition (HAI) assay using turkey red blood cells (RBC) and neutralization assay using MDCK cells as described previously [53], [55]. Ferrets were considered to have seroconverted if the antibody titer was higher than the limit of detection. The limit of detection is 1∶10 for the HAI assay and 1∶20 for the neutralization assay.

### Aerosol Particle Sampling

Aerosol sampling of the ferret cages was performed between 9 am and 12 pm on alternate days for 10 days, prior to nasal wash collection. The air samples were collected by placing cyclone-based air samplers (BC251) developed by the National Institute for Occupational Safety and Health (Morgantown, WV) [22] on the outside of the infected side of the ferret transmission cage. A designated air sampler was used for each ferret to reduce cross-contamination between animals. A baseline or day 0 reading was obtained on the infected ferrets prior to inoculation and 24 hrs after the animal was placed into the transmission cage. Air was sampled for one hour at a flow rate of 3.5 liters per minute. The aerosol sampler flow rate was calibrated before each use using a flow meter (TSI 4100 series). The NIOSH BC251 samplers separate particles based upon size. Each sampler contained an empty 15 mL conical that collected particles greater than 4 µm, a 1.5 mL conical that collected particles between 1–4 µm, and a 3 µm pore Fluoropore membrane filter (Millipore) to collect submicron particles.

Processing of the samplers was performed in a bio-safety cabinet; 500 µL of Ambion RNA lysis binding buffer was placed into each collection tube and vortexed vigorously. RNA was extracted from each collection tube on a QIAGEN EZ Robot using the QIAGEN EZ1 virus mini kit, per the manufacturer's recommendations. The total amount of influenza RNA was quantified using Applied Biosystems Taqman one-step RT-PCR kit with primers (F – 5′AGATGAGTCTTCTAACCGAGGTCG3′, and R - 5′GCAAAGACATCTTCAAGTCTCTG3′) and a probe (FAM-TCA GGC CCC CTC AAA GCC GA–[NFQ]) specific for the influenza A M gene segment. In vitro transcribed RNA corresponding to this region of the M gene segment was used as a standard for absolute quantification. The RNA standard was created by, linearizing a pCDNA3.1 plasmid containing the T7 promoter and M gene sequences of influenza virus strain A/Beijing/262/95. This DNA was used as templates with the MEGAScript In Vitro Transcript Kit (Ambion) to generate Flu A M gene transcript. Transcripts were purified by extraction with Phase Lock Gel (PLG) (Heavy) tubes (Eppendorf Scientific, Inc.) two times, followed by phenol/chloroform, chloroform extraction and ethanol precipitation. The dried RNA pellet was resuspended in RNase-free RNA storage buffer (1 mM sodium citrate, pH 6.4; Ambion). The concentration of the purified transcript was determined by measuring absorbance at 260 nm. 10-fold serial dilution of the FluAM transcript in RNA storage buffer was performed to generate transcript at the level of 5×10^6^ down to 5 copies/µL.

The limit of detection of the NIOSH BC251 samplers is unknown. After each use, the BC251 samplers were decontaminated by first rinsing each sampler with distilled water, making sure to wash the air inlet and other holes, then washing the samplers with isopropanol, again running the alcohol through the air inlet and all other holes.

### Neuraminidase Activity

Activity of the NA protein of each virus was determined using a peanut-agglutinin based enzyme-linked lectin assay (ELLA). The ELLA assay was slightly modified from a previously described assay [56]. The ELLA assay uses fetuin as a substrate for the viral neuraminidase. Viruses were normalized for infectivity, 10^6.5^ TCID_50_ per 500 µL, prior to performing the assay. Neuraminidase activity using MUNANA as a substrate was preformed using the NA star kit obtained from Applied Biosystems, and following the manufacturers instructions.

### Electron Microscopy for Virus Morphology

A 2 mL aliquot of each stock virus, described in the ‘Viruses’ section, was concentrated by ultra-centrifugation using a Beckman Coulter L-100 XP ultracentrifuge with a SW-55i rotor. Viruses were pelleted at 24K rpm for 2 hrs; the pellet was resuspended in 20 µL of 1x Karnovsky's fixative solution. Concentrated viruses were sent to the Electron Microscopy unit at Rocky Mountain Laboratory (Hamilton, MT) for negative stain and analysis. Freshly glow discharged Formvar-carbon coated copper grids (Ted Pella, Inc., Redding, CA) were submerged in droplets of each sample and incubated overnight at 4 degrees C in a humid chamber. The grids were washed three times for 5 min each in deionized water, and negatively stained for 15 sec with methylamine tungstate (Nanoprobes, Inc., Brookhaven, NY). The grids were examined at 80 kV on a Hitachi H7500 transmission electron microscope. Digital images were captured on an HR-100 CCD camera (Advanced Microscopy Techniques, Danvers, MA), and rendered using Adobe PhotoShop (Adobe Systems, Inc., San Jose, CA). The percent filamentous particles were calculated by counting over 20 particles for each virus from blind pictures taken randomly on the grid.

### Supplemental Methods

#### In vitro replication kinetics

MDCK cells were infected with each virus at an MOI of 0.1 and supernatant from infected cells in triplicate was collected at 8, 24, 30, and 48 hours post-infection. Supernatants were titrated on MDCK cells by serial dilution as previously described [57].

#### In vivo replication kinetics

Replication of viruses in the upper and lower respiratory tract of 8–12 wk old ferrets was determined as previously described [53]. Each ferret was inoculated IN with 10^6^ TCID_50_ of virus in 500 µL. Nasal turbinates and lung sections were harvested on 1 and 5 days post infection. Viral titers in each organ were determined as previously described [57].

#### Influenza receptor binding assay

Receptor specificity was determined using an in vitro receptor binding assay as described previously [58]. Chicken RBCs (Lampire Biological Laboratories Inc) were desialylated with *Clostridium perfringens* neuraminidase (SIGMA). The desialylated RBC were resialylated using specific α2,3 (SIGMA) or α2,6 (Calbiochem) sialyltransferases. Viruses known to bind specifically to α2,3- and α2,6-linked sialic acids were used as controls for each experiment.

#### Receptor affinity assay

The affinity of a virus was determined as previously described [45]. Briefly, chicken RBCs (Lampire Biological Laboratories Inc) were treated with serial dilutions of *Clostridium perfringens* neuraminidase (SIGMA) to remove sialic acids. Agglutination of RBCs treated with the different neuraminidase concentrations was determined using a standard amount of each virus (4 HAU).

## Supporting Information

Figure S1 **The Rec pH1N1 virus behaves like the biological pH1N1.** Ferrets, 6–8 weeks old, were infected with either Rec pH1N1 or biological pH1N1. Virus titers were measured on days 1 and 5 post infection in the nasal turbinates (A) or lung (B). MDCK cells were infected with biological or Rec pH1N1 and virus titers were determined at the time indicated (C). Transmission efficiency of the biological pH1N1 virus was determined using 3 transmission cages with 6 adult ferrets (D).
(TIFF)

Figure S2 **Reduced transmission and release of particles containing influenza viral RNA from ferrets infected with TRS virus.** Six ferrets were inoculated IN to test the RD transmission of TRS. Nasal washes were collected on the indicated days (A). Each bar represents the titer of virus from an individual ferret. Inf stands for infected ferret. The limit of detection is represented as the dashed line and is 10^0.5^ TCID_50_ per mL. Serum was collected on day 0 and day 14. Anti-influenza antibodies were measured by HAI and neutralization assay (B). The limit of detection is 1∶10 for HAI and 1∶20 for the neutralization assay. Antibody titers in the day 0 sera were below the limit of detection. Aerosol sampling was performed on four of the infected animals (Inf 1–4) to determine the presence of particles containing influenza viral RNA (C). Each bar represents an individual animal. Absolute RNA was quantified using a standard curve of in vitro transcribed influenza M gene RNA.
(TIFF)

Figure S3 **Schematic of respiratory droplet transmission cage setup.** Commercially available cages from Allentown were modified to prevent direct contact between the two ferrets. A top-down view of the modified cage illustrates the location of the infected and naïve ferret in relation to the airflow (A). A door containing separate water and feeding tray for each ferret (B) and a perforated stainless steel panel (C) prevented any contact between the ferrets.
(TIFF)

Figure S4 **The 2009 pandemic H1N1 virus and precursors share receptor specificity and affinity.** An in vitro receptor-binding assay using desialylated chicken RBCs was used to determine the receptor binding of the Rec pH1N1, 6∶2 reassortant, TRS, and Eurasian swine viruses (A). Viruses with differential receptor specificity, previously identified by MedImmune, were used as controls in the receptor-binding assay. The α2,3 standard is A/Japan/305/1957 (H2N2) Q226, G228 and the α2,6 standard is A/Japan/305/1957 (H2N2) L226, S228. Receptor affinity was assessed by agglutination of partially desialylated RBCs (B). Viruses defined previously to have differential receptor affinity [59] were used as standards.
(TIFF)

Table S1 **Summary of clinical signs in infected and naïve ferrets.**
(DOC)
