# Supplementary figures and images for: Eurasian-Origin Gene Segments Contribute to the Transmissibility, Aerosol Release, and Morphology of the 2009 Pandemic H1N1 Influenza Virus
Source: PLoS Pathog. 2011 Dec 29;7(12):e1002443. doi: 10.1371/journal.ppat.1002443 (PMC3248560; doi:10.1371/journal.ppat.1002443)

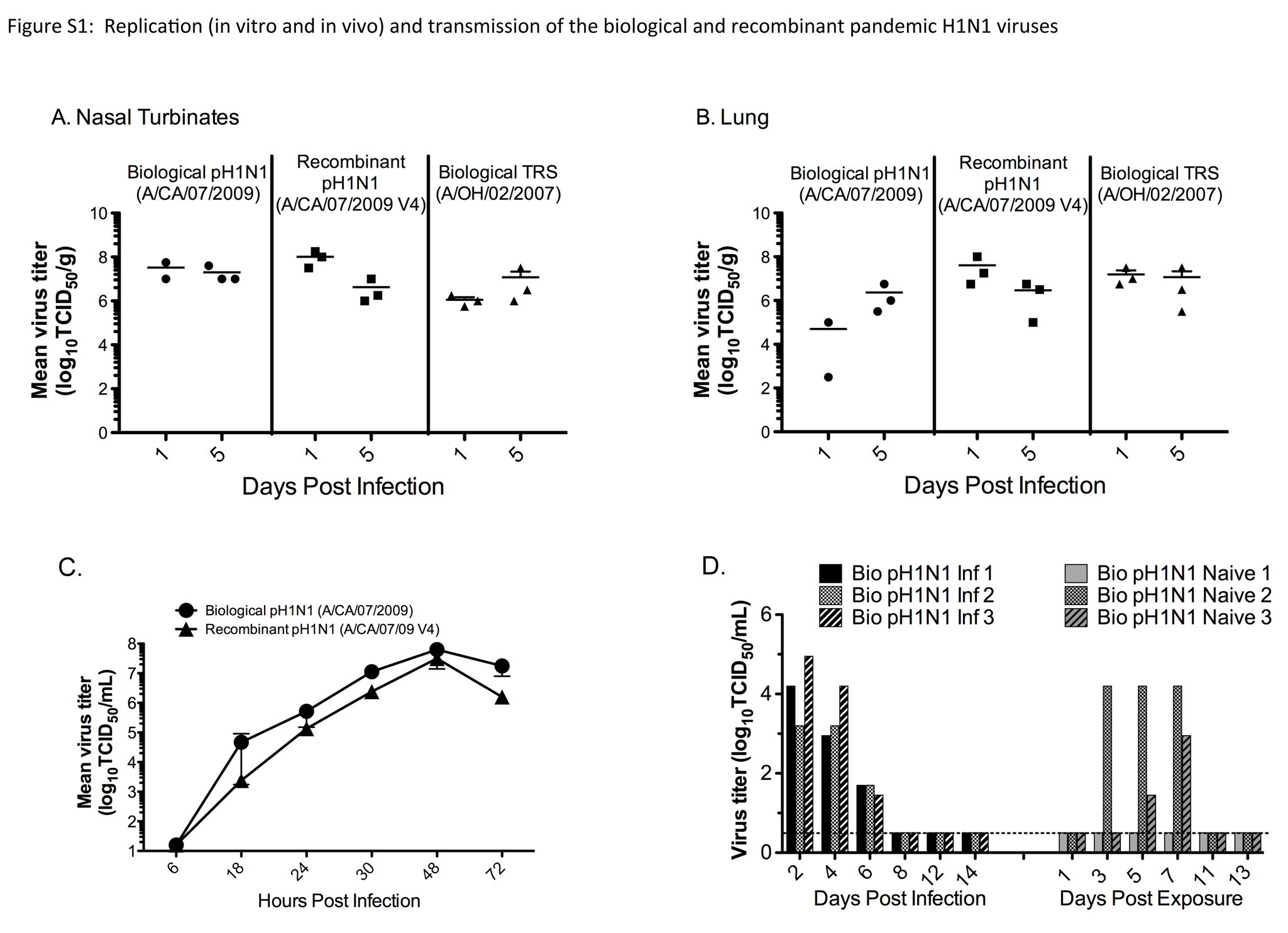

Supplement: Figure S1 — The Rec pH1N1 virus behaves like the biological pH1N1. Ferrets, 6–8 weeks old, were infected with either Rec pH1N1 or biological pH1N1. Virus titers were measured on days 1 and 5 post infection in the nasal turbinates (A) or lung (B). MDCK cells were infected with biological or Rec pH1N1 and virus titers were determined at the time indicated (C). Transmission efficiency of the biological pH1N1 virus was determined using 3 transmission cages with 6 adult ferrets (D). (TIFF) [file ppat.1002443.s001.tiff]

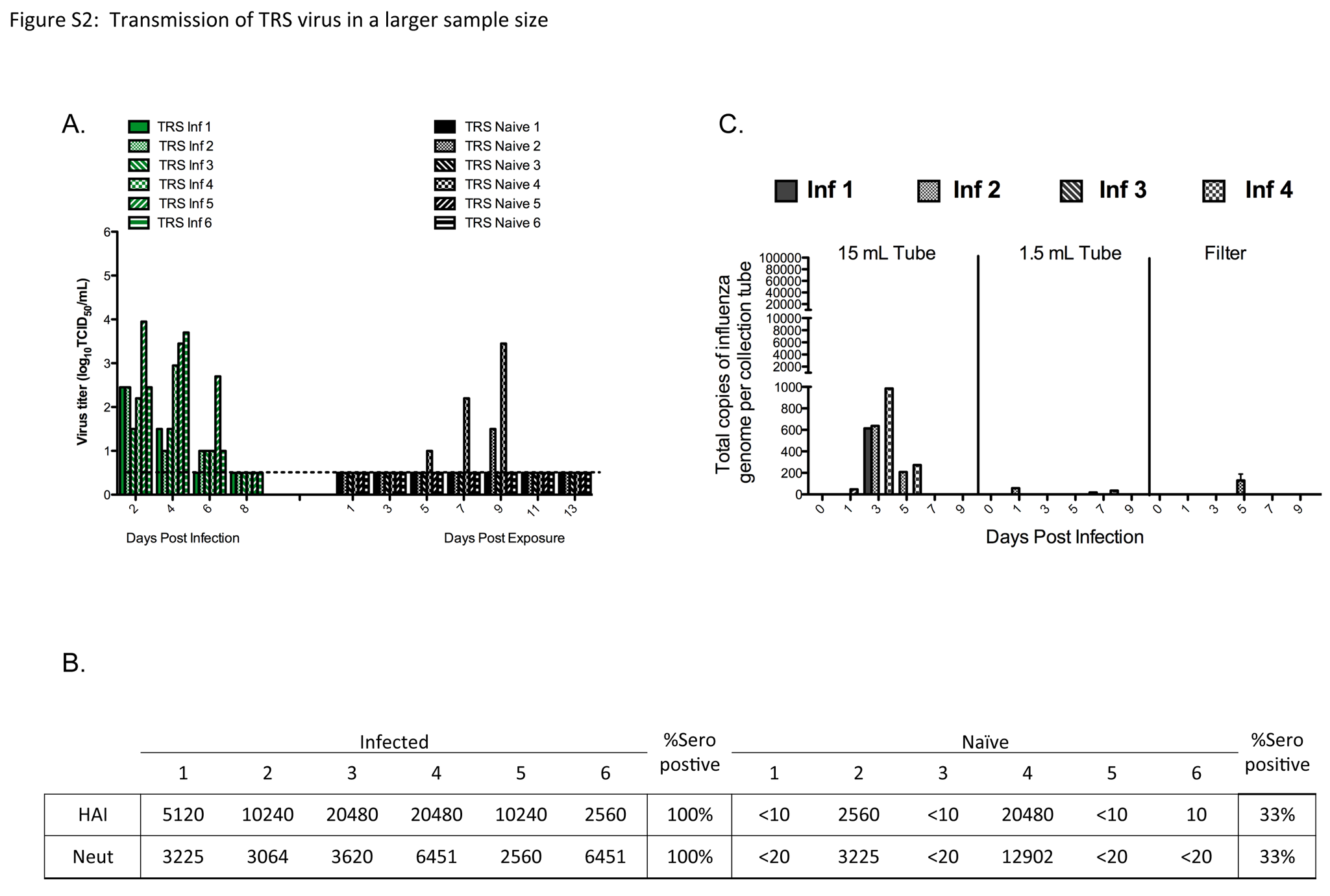

Supplement: Figure S2 — Reduced transmission and release of particles containing influenza viral RNA from ferrets infected with TRS virus. Six ferrets were inoculated IN to test the RD transmission of TRS. Nasal washes were collected on the indicated days (A). Each bar represents the titer of virus from an individual ferret. Inf stands for infected ferret. The limit of detection is represented as the dashed line and is 100.5 TCID50 per mL. Serum was collected on day 0 and day 14. Anti-influenza antibodies were measured by HAI and neutralization assay (B). The limit of detection is 1∶10 for HAI and 1∶20 for the neutralization assay. Antibody titers in the day 0 sera were below the limit of detection. Aerosol sampling was performed on four of the infected animals (Inf 1–4) to determine the presence of particles containing influenza viral RNA (C). Each bar represents an individual animal. Absolute RNA was quantified using a standard curve of in vitro transcribed influenza M gene RNA. (TIFF) [file ppat.1002443.s002.tiff]

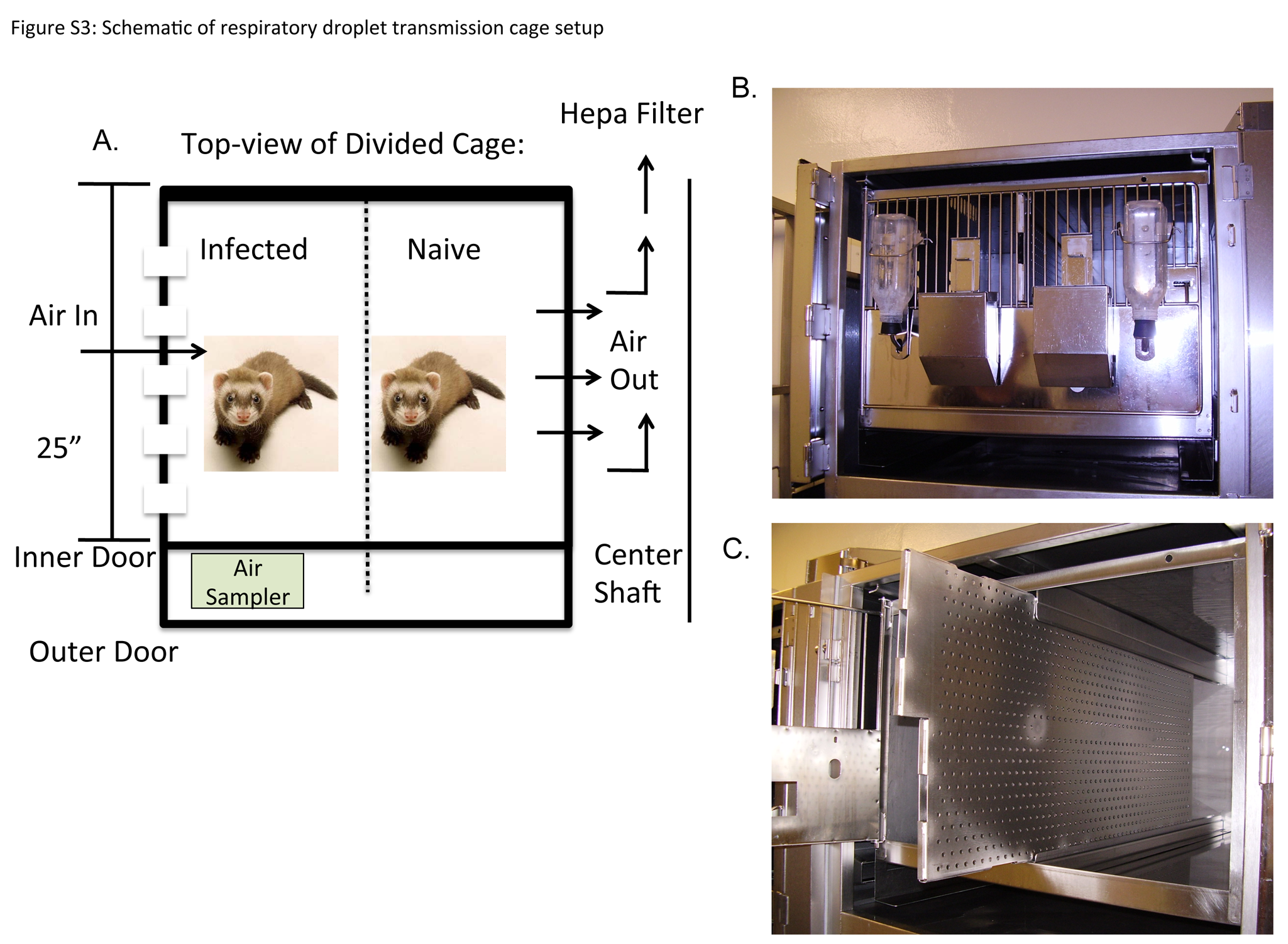

Supplement: Figure S3 — Schematic of respiratory droplet transmission cage setup. Commercially available cages from Allentown were modified to prevent direct contact between the two ferrets. A top-down view of the modified cage illustrates the location of the infected and naïve ferret in relation to the airflow (A). A door containing separate water and feeding tray for each ferret (B) and a perforated stainless steel panel (C) prevented any contact between the ferrets. (TIFF) [file ppat.1002443.s003.tiff]

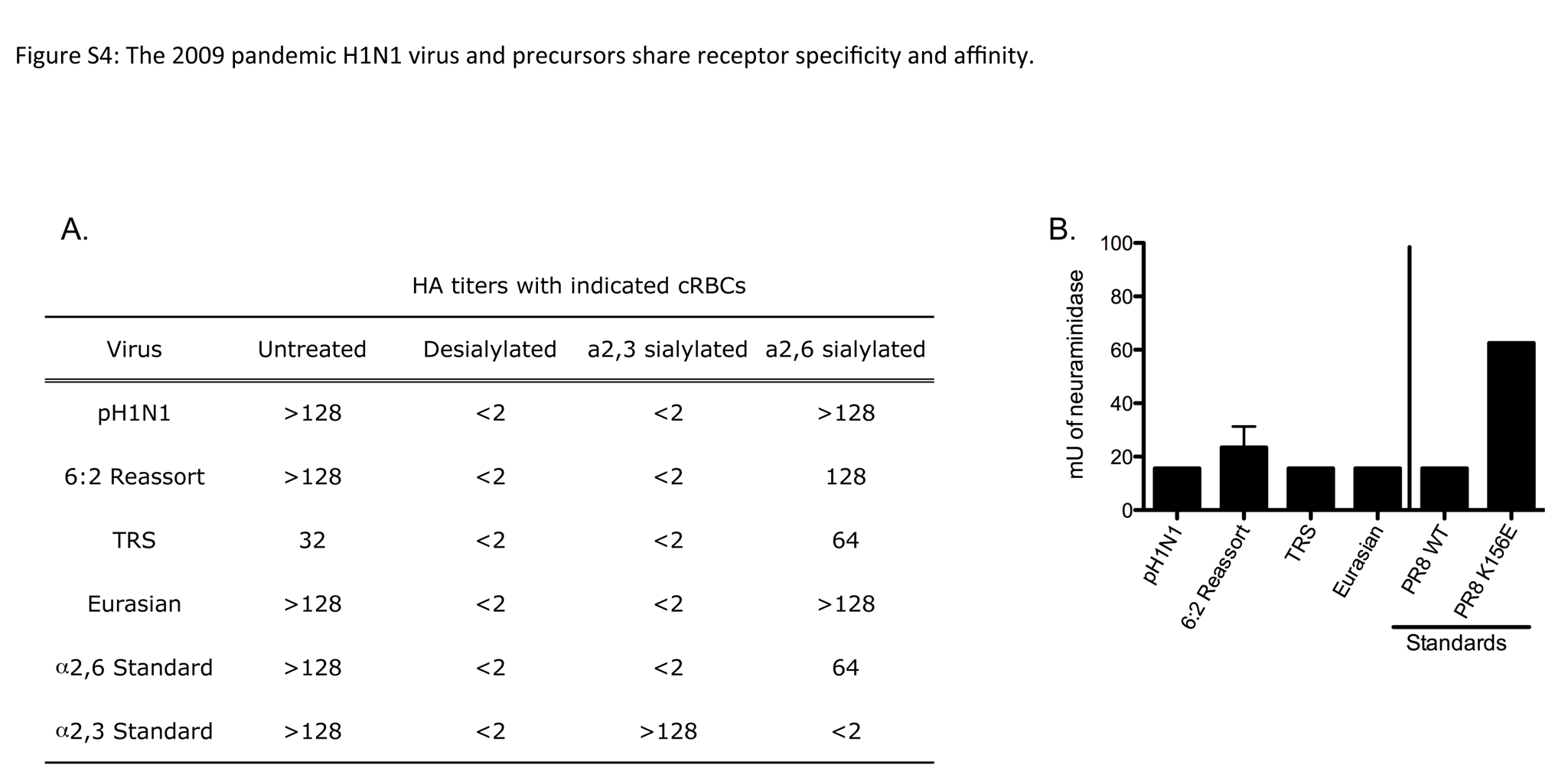

Supplement: Figure S4 — The 2009 pandemic H1N1 virus and precursors share receptor specificity and affinity. An in vitro receptor-binding assay using desialylated chicken RBCs was used to determine the receptor binding of the Rec pH1N1, 6∶2 reassortant, TRS, and Eurasian swine viruses (A). Viruses with differential receptor specificity, previously identified by MedImmune, were used as controls in the receptor-binding assay. The α2,3 standard is A/Japan/305/1957 (H2N2) Q226, G228 and the α2,6 standard is A/Japan/305/1957 (H2N2) L226, S228. Receptor affinity was assessed by agglutination of partially desialylated RBCs (B). Viruses defined previously to have differential receptor affinity [59] were used as standards. (TIFF) [file ppat.1002443.s004.tiff]
