# Supplementary material for: Eurasian-Origin Gene Segments Contribute to the Transmissibility, Aerosol Release, and Morphology of the 2009 Pandemic H1N1 Influenza Virus
Source: PLoS Pathog. 2011 Dec 29;7(12):e1002443. doi: 10.1371/journal.ppat.1002443 (PMC3248560; doi:10.1371/journal.ppat.1002443)
Supplement: Table S1 — Summary of clinical signs in infected and naïve ferrets. (DOC) [file ppat.1002443.s005.doc]

**Table S1: Summary of Clinical Signs in Infected and Naïve Ferrets**

|  |  |  | Clinical Signs | | | | |
| --- | --- | --- | --- | --- | --- | --- | --- |
| Virus | Status | Transmission Efficiency | Temperature Increase^a^ | Weight loss >10% | Reuman Nasal Score^b^ No. pos/total (score) | Reuman Activity Score^c^  No. pos/total (score) | Other Clinical Signs |
| Rec pH1N1 | Infected |  | 2/4 | 2/4 | 1/4 (2) | 1/4 (1) |  |
|  | Naïve | 4/4 | 2/4 | 2/4 | 0 | 2/4 (2,2) |  |
| 6:2 Reassort | Infected |  | 0/4 | 3/4 | 1/4 (1) | 0 |  |
|  | Naïve | 2/4 | 1/4 | 0/4 | 1/4 (1) | 0 |  |
| TRS | Infected |  | 1/4 | 0/4 | 2/4 (1,1) | 0 | Croup in 1/4 animals |
|  | Naïve | 2/4 | 2/4 | 0/4 | 1/4 (1) | 0 |  |
| Eurasian | Infected |  | 1/4 | 1/4 | 2/4 (1,1) | 0 | Croup in 1/4 animals |
|  | Naïve | 2/4 | 0/4 | 0/4 | 1/4 (1) | 0 |  |

a - Temperature change greater than 1.5 degrees from day 0 temp

b - Reuman scores [49]: 1 - nasal rattling, sneezing, or coughing; 2 - nasal discharge on their external nares; 3 - mouth breathing.

c – Reuman scores [49]: 0- fully playful, 1-did not initiate play, 2 – alert but not playful, 3 – neither alert nor playful

The number in parentheses indicates the Reuman score of ferrets that experienced a symptom.
